# Benefit from regular versus leakage-related exchange of voice prostheses in patients post-laryngectomy considering complication rates and patient satisfaction feedback—a randomized case−controlled trial

**DOI:** 10.3389/fonc.2025.1468955

**Published:** 2025-01-24

**Authors:** Michał Żurek, Małgorzata Czesak, Daniel Majszyk, Anna Rzepakowska

**Affiliations:** Otorhinolaryngology Department, Head and Neck Surgery, Medical University of Warsaw, Warsaw, Poland

**Keywords:** voice prosthesis, voice rehabilitation, total laryngectomy, prosthetic leakage, replacement rate, device lifetime

## Abstract

**Objective:**

The complications related to voice prosthesis usage substantially affect the physical and social functioning of patients after total laryngectomy, which influences their quality of life. Leakage dysfunction is the most common, causing uncertainty and requiring unscheduled hospital visits. Our study was designed to estimate the benefit of regular versus leakage-related replacement of the voice prosthesis. Study Design. Randomized case−controlled trials. Setting. Tertiary hospital.

**Methods:**

The study included patients who underwent total laryngectomy with primary voice prosthesis insertion between 2020 and 2021 and were randomly assigned to one of two arms: regular exchange (REA) every 3 months or leakage-dependent exchange (LEA). The control treatment was continued for 12 months. The primary outcome measure was the comparison of complication rates in both arms, including periprosthetic leakage, granulation or atrophy of mucosa around the fistula, and dislocation of the prosthesis. The secondary outcome measures were the mean number of exchanges per year and patient satisfaction.

**Results:**

Thirty-six patients continued the study according to the protocol, with 16 in REA and 20 in LEA. A total of 153 voice prostheses were replaced during the study period, including 98 in REA and 55 in LEA. Comparative analysis of REA and LEA revealed a significantly longer time between replacements in the LEA group (p = 0.023) and a significantly lower rate of complications in the REA group (p = 0.029). Periprosthetic leakage was the most common complication associated with the use of voice prostheses, occurring in 3.06% of patients in REA and 9.09% in LEA, but this difference was statistically insignificant (p = 0.137). The analysis of factors predisposing patients to leakage failure revealed that treatment schemes, concomitant diseases, dental conditions, and diet or alcohol consumption significantly affect the longevity of voice prostheses. The relative and absolute risks (RRR and ARR) of complications in the REA group were reduced by 69.39% and 13.88%, respectively. The number of replacements (NNTs) that should be performed in the REA scheme to prevent one complication over the LEA scheme is 721.

**Conclusion:**

The replacement of regular voice prostheses improved the overall complication rate over the scheme based on leakage demand; however, it did not yield superior benefits in terms of patient satisfaction or economic aspects.

**Clinical Trial Registration:**

https://clinicaltrials.gov/study/NCT04268459, identifier NCT04268459.

## Introduction

The tracheoesophageal voice with voice prosthesis is currently the mainstay of voice rehabilitation post laryngectomy (1). The uncomplicated technique of tracheoesophageal fistula formation with the insertion of a prosthesis during laryngectomy, followed by the quick and easy process of voice rehabilitation, is the main encouraging factor. However, the use of a prosthesis is associated with a significant number of complications, ranging from 10 to 60%. The most common reported complication is transprosthetic leakage (55–80%), which determines the need for device exchange (2–5). However, some patients experience more serious problems, e.g., periprosthetic leakage (5–30%), granulation or atrophy of the mucosa around the fistula, and dislocation of the prosthesis, which may require anti-inflammatory treatment, temporary nasogastric tube feeding or surgical procedures. The standard protocol is voice prosthesis exchange due to transprosthetic leakage. Optionally, the device can be replaced regularly to prevent both transprosthetic leakage and other complications. The majority of prostheses are made of silicone and are placed in conditions exposed not only to chemical and biological compounds contained in the ingesta and patient secretions but also to mechanical forces related to speech, swallowing, and coughing. Like any device, they have a limited lifespan, depending not only on the technology and type of prosthesis but also on host-related factors, including proper hygiene and cleaning, biofilm formation, and infection (6). Most of the currently used voice prostheses are indwelling prostheses, which are categorized as normal or problem-solving. Primary insertion is performed with the normal category. The so-called problem-solving voice prosthesis is indicated when frequent replacement of the normal prosthesis is necessary due to complications. The expected median lifetime of a normal prosthesis ranges from 2–6 months (6, 7), whereas the median lifetime of a problem-solving device is 337 days (8). However, the cost of the problem-solving prothesis is five times greater.

Total laryngectomy is a handicapping procedure that significantly affects patients’ postoperative quality of life. The voice prosthesis surely improves communication and reduces communication limitations. However, unexpected dysfunction of the device affects the sense of comfort. A waiting attitude with the risk of unexpected leakage may significantly disorganize patients’ lives. On the other hand, the exchange procedure, although performed as a one-day hospitalization or outpatient procedure, is inevitably associated with certain discomfort for the patient. There has not been any consensus on the scheme of prosthesis replacement thus far, although studies more frequently question the rationale for regular exchanges and search for biological or immunological reasons to support the right concept (9).

In this prospective study, we plan to compare the benefits of regular (every three months) versus leakage-related exchanges of voice prostheses after laryngectomy in terms of the rate of complications and patient feedback.

## Materials and methods

The study has been reported in Clinical Trial (NCT04268459) under the title Estimation of Benefit From Regular Versus Leakage-related Exchange of Voice Prosthesis in Patients Post Laryngectomy. It was also approved by the Bioethics Committee at the Medical University of Warsaw (No KB/58/2020). All patients were informed about the objectives of the study and signed informed consent forms. The study included all patients with laryngeal cancer who underwent total laryngectomy with primary insertion of voice prostheses between January 2020 and December 2021 in our center. The exclusion criteria were laryngopharyngectomy with digestive tract reconstruction with a free flap jejunal or surgery at another center. Other demographic and clinical factors were not considered among the inclusion and exclusion criteria. Patients were randomly assigned to two groups via the block randomization method. The aim of choosing such a method was to obtain equal groups of patients. The block consisted of two patients, one of whom was randomly allocated to each group. The allocation indicated that one of the two arms of the study and that postoperative management influenced the recommendations of care. The first group of patients was referred to the regular exchange arm, and they were appointed every 3 months for regular exchange of voice prosthesis (REA – regular exchange arm). The second group was the leakage exchange arm (LEA–leakage exchange arm), which was instructed to appear in the department if voice prosthesis leakage or other complications were noticed. The laryngectomy with primary insertion and the prothesis exchanges were performed randomly by one out of three ENT specialists. The study was completed in March 2023. A follow-up period of at least 12 months after laryngectomy was assumed for each patient. Voice prosthesis exchange in all patients was performed via the same scheme under local anesthesia, and the same queries from the protocol were checked and fulfilled. The primary outcome measure was the comparison of complication rates in both arms: periprosthetic leakage, granulation or atrophy of the mucosa around the fistula, and dislocation of the prosthesis. The secondary outcome measures were the mean number of exchanges per year and patient satisfaction in both arms. For each voice prosthesis exchange, patients were asked three questions assessed with the visual analogue scale (VAS), concerning their feedback on voice prosthesis use, nuisance related to the procedure of prosthesis replacement and self-assessed voice quality. In addition, a medical history was collected regarding primary and adjuvant treatment, as well as concomitant diseases that were selected as relevant to the study (diabetes, gastroesophageal reflux, peptic ulcer disease, COPD, asthma). Patients also completed questionnaires about their habits (HME, cleaning VPs, stimulants, diet).

Statistical analysis was performed with IBM SPSS Statistics (version 29.0). The statistical significance level was fixed at 0.05. Descriptive statistics of the study group were calculated. Differences between numerical variables stratified by nominal variables were evaluated via the Mann−Whitney U test and the Kruskal−Wallis test. Differences in the distributions of nominal outcomes were analyzed via chi-square tests. The gain from regularity of voice prosthesis replacements was assessed via the following indicators: relative, absolute risk reduction and number needed to treat. The variability in vocal prosthesis lifetime was assessed via inter- and intrapatient coefficients of variability.

## Results

The study group included 40 patients who underwent total laryngectomy (TL) with primary insertion of voice prostheses and were randomly assigned to both REA and LEA study groups. The final evaluation after 12 months revealed that 36 patients continued according to the protocol, with 16 in REA and 20 in LEA. The majority of patients were male (75%), including 12 with REA and 15 with LEA (p= 0.99). The mean age of patients at the time of laryngectomy in the REA was 69 years (± 7.79), which was significantly greater (p=0.002) than that in the LEA [60 years (± 8.17)].

The body mass and height were comparable for both groups. The mean weight of patients in the REA group was 72.63 kg (± 16.96), and that of patients in the LEA group was 68.15 kg (± 12.88) (p=0.374). The average height of patients in the REA was 169.31 cm (± 7.51), whereas that in the LEA was 170.05 cm (± 9.98) (p=0.81). Almost all patients in the study group had a history of smoking, 14 in REA and 19 in LEA (p=0.84). Only 7 patients denied alcohol consumption, including 5 in REA and 2 in LEA (p=0.28).

All patients had advanced-stage laryngeal cancer (T3, T4a) at the time of diagnosis, and in the majority of cases, TL was the primary treatment option for 15 patients from both REA and LEA (p= 0.294). All patients were referred to a speech therapist in the postoperative period, and they received comprehensive instructions on hygiene and maintenance of the prosthesis. Patient characteristics are shown in **Table 1**.

**Table 1:** Patient characteristics in the regular and leakage exchange arms.

Characteristic		Regular exchange arm (REA) n=16	Leakage exchange arm (LEA) n=20	p value
Gender	Female	4	5	0.999
	Male	12	15	
Age in years at the time of TL		68.75 ± 7.79	59.85 ± 8.17	**0.002**
Weight (kg)		72.63 ± 16.96	68.15 ± 12.88	0.374
High (cm)		169.31 ± 7.51	170.05 ± 9.98	0.808
BMI		25.14 ± 4.46	23.16 ± 3.15	0.129
Smoking	No	2	1	0.84
	Yes	14	19	
	Pack-years	41.43 ± 20.98	37.63 ± 11.47	0.843
Alcohol consumption	No	5	2	0.276
	Occasionally	9	15	
	Often	2	3	
Education	Primary	7	10	0.281
	Secondary	7	9	
	Higher	2	0	
Habitation	Country	1	7	0.097
	Town	15	13	
Primary treatment	Surgery	15	15	0.294
	Radiotherapy	1	5	
Adjuvant therapy	None	3	8	0.329
	Radiotherapy	10	8	
	Radiochemotherapy	3	4	

TL, total laryngectomy; BMI, body mass index.

Bold values indicates p- value < 0.05.

A total of 153 voice prostheses were replaced during the study period, including 98 in REA and 55 in LEA. The average replacement time for a voice prosthesis in REA was 3.03 months (SD ± 1.01), whereas that in LEA was 5.37 months (SD ± 4.74). The Mann−Whitney U test revealed a significant difference in the time of replacement between the groups (p=0.023).

Considering all the identified complications, we found a significantly greater rate in the LEA group than in the REA group (11 versus 6, respectively) (p=0.023). The analysis of individual types of problems was, however, indifferent. The most common complication was leakage around the prosthesis (47.1%), with 3 patients in REA and 5 in LEA; however, there was no significant difference between the two arms (p=0.121). In addition to periprosthetic leakage, other complications, including the formation of granulation tissue around the voice prosthesis, displacement of the device and atrophy of the mucosa around the fistula difficulties in replacing the voice prosthesis, were reported in 9 patients, with 6 in each group. **Table 2** summarizes the procedures of prothesis exchanges in REA and LEA.

**Table 2:** Exchanges of voice protheses in REA and LEA.

Characteristic	REA	LEA	p value
Number of VP replacement	98	55	
Average replacement time (months)	3.03 ± 1.01	5.37 ± 4.74	**0.023**
Complications	6 (6.12%)	11 (20%)	**0.029**
Leakage around the prothesis	3 (3.06%)	5 (9.09%)	**0.137**
Granulation tissue	1 (1.02%)	3 (5.45%)	0.262
Displacement of VP	2 (2.04%)	1 (1.82%)	0.924
Mucosal atrophy	0 (0%)	2 (3.64%)	0.247
Difficult replacement	6 (6.12%)	6 (10.91%)	0.103

REA, regular exchange arm group; LEA, leakage exchange arm group; VP, voice prothesis.

Bold values indicates p- value < 0.05.

All patients at each voice prosthesis replacement rated their satisfaction with the use of the voice prosthesis on an 11-point visual analogue scale (VAS) (0, extremely unsatisfied; 10, very satisfied), and the mean score was comparable between the two arms, with scores of 6.99 for REA (SD ± 2.89) and 6.65 for LEA (SD ± 2.82) (p = 0.389). The evaluated nuisance of the voice prosthesis replacement with the VAS (0 – not at all and 10 – very uncomfortable) was rated 0.77 (SD ± 1.86) and 1.02 (SD ± 2.29), respectively, for REA and LEA (p = 0.9). Patients rated their voices on the VAS scale (0–extremely dissatisfied and 10–very satisfied), and the REA obtained a mean score of 5.07 (SD ± 3.71) compared with 5.69 (SD ± 3.53) for LEA (p =0.337).

In the LEA, the factors affecting prosthesis lifetime were examined. The difference in lifetime between females and males was not significant (p=0.72), with values of 4.96 months and 5.72 months, respectively. The replacements in LEA were divided by the age of the patients at total laryngectomy: 43 were under 65 years of age (LEA1), and 12 were 65 years or older (LEA2). The age of the patients had no statistically significant effect on the lifetime of the device; the prosthesis was replaced every 4.9 months (SD ± 4.12) in patients in LEA1 and every 7.08 months (SD ± 6.42) in LEA2, p=0.192. There were 27 exchanges in patients with primary education every 5.61 months (SD ± 4.68) and 16 exchanges in patients with secondary education every 6.5 months (SD ± 5.8), p=0.771. Moreover, the place of residence of the patients did not affect the lifetime of the prosthesis (p=0.809). The prosthesis was replaced every 5.43 months (SD ± 4.78) in 21 patients living in rural areas and every 5.62 months (SD ± 4.99) in 30 patients living in towns. Regardless of the presence of chronic diseases in patients, the survival time of the prosthesis was 5 months; in 17 patients with chronic diseases, it was 5.58 months (SD ± 5.51); and in 38 patients without comorbidities, it was 5.27 months (SD ± 4.42) (p=0,847). Furthermore, the active use of stimulants in the form of smoking or alcohol consumption by the patients did not affect the survival time of the voice prostheses, p=0.307 and p=0.367, respectively. We checked whether oral hygiene impacts the time between replacements. The survival time of the voice prosthesis was 5.43 months (SD ± 3.97) in 15 patients with their own teeth, 7.69 months (SD ± 6.79) in 13 patients with dentures, and only 4.22 months (SD ± 3.58) in 27 patients with caries; however, the differences were not significant (p=0.154). A diet rich in fruits and vegetables provided by 19 patients did not affect the device survival time, which was 5.92 months (SD ± 5.01) compared with 5.14 months (SD ± 4.69) for 35 patients who denied a varied diet (p=0.635).

Patients reported cleaning the prosthesis with a brush during 47 exchanges, whereas in 7 exchanges, they denied it. The prosthesis lifetimes were 4.88 months (SD ± 3.7) and 7 months (SD ± 8.31), respectively (p=0.999). The survival time of the prosthesis was 7.41 months (SD ± 5.91) when heat and moisture exchangers (HME) were not used for 23 replacements and 3.96 months (SD ± 2.42) when HME was used for 25 replacements (p=0.073). Provox Vega was implanted in 51 patients, and Provox ExtraSeal was implanted in 4 patients; however, the prosthesis type did not affect the time between replacements, which was 5.6 months (SD ± 4.84), and 2.5 months (SD ± 1) (p=0.221). Primary surgical treatment by TL was performed as salvage surgery in 19 patients, but it did not affect the time between device replacements—6.26 months (SD ± 5.27) and 3.68 months (SD ± 2.94), respectively. Adjuvant treatment, RT in 24 patients and RCT in 5 patients, revealed a significant difference in the lifetime of the device (p=0.032). It was especially prolonged in patients in the RCT (10.1 months, SD ± 5.15). **Table 3** shows the average time between voice prothesis replacements according to the selected factors and their statistical significance.

**Table 3:** Factors affecting prosthesis longevity in the LEA group included 55 replacements (some data were collected via questionnaires completed by patients).

Variable		No. of replacements	Average time to exchange (in months)	P value
Sex	Woman	12	4.96 ± 4	0.72
	Man	43	5.72 ± 5.12	
Age at surgery	>=65 lat	12	7.08 ± 6.42	0.192
	<65 lat	43	4.9 ± 4.12	
Education	Primary	27	5.61 ± 4.68	0.771
	Secondary	16	6.5 ± 5.8	
Place of residence	Rural	21	5.43 ± 4.78	0.809
	Urban	30	5.62 ± 4.99	
Regular cleaning with brush	Yes	47	4.88 ± 3.7	0.999
	No	7	7 ± 8.31	
Regular HME using	Yes	25	3.96 ± 2.42	0.073
	No	23	7.41 ± 5.91	
Type of prosthesis	Vega	51	5.6 ± 4.84	0.221
	Extra Seal	4	2.5 ± 1	
Primary treatment	Surgery	36	6.26 ± 5.27	0.053
	Others	19	3.68 ± 2.94	
Adjuvant treatment	RT	24	6.08 ± 5.67	**0.032**
	RCT	5	10.1 ± 5.15	
	None	26	3.81 ± 2.67	
Comorbidities	Present	17	5.58 ± 5.51	0.847
	Absent	38	5.27 ± 4.42	
Oral cavity	Healthy teeth	15	5.43 ± 3.97	0.154
	Dentures	13	7.69 ± 6.79	
	Caries	27	4.22 ± 3.58	
Smoking	Currently	10	4.15 ± 3.84	0.307
	In the past	45	5.66 ± 4.96	
Alcohol consumption	Yes	26	5.31 ± 4.43	0.367
	No	19	6.87 ± 5.73	
Diet rich in vegetables and fruits	Yes	19	5.92 ± 5.01	0.635
	No	35	5.14 ± 4.69	

Missing data in the analysis are due to incorrectly completed questionnaires.

HME, heat and moisture exchanger; RT, radiotherapy; RCT, radiochemotherapy.

Bold values indicates p- value < 0.05.

An analysis of the variability in the device lifetime during LEA was also performed. The interpatient coefficient of variability (CV) was 65.79%, indicating strong device lifetime variability among all patients. The intrapatient CV was 53.65%, indicating strong, although slightly lower, average variability in the replacement interval among each patient individually.

The last table (**Table 4**) shows the results of evaluating the effectiveness of regular over irregular replacements in terms of the emergence of complications. The relative and absolute risks (RRR and ARR) of complications in the REA group were reduced by 69.39% and 13.88%, respectively. The number of replacements (NNTs) that should be performed in the REA scheme to prevent one complication over the LEA scheme is 721.

**Table 4:** Comparison of complication assessment parameters between REA and LEA.

Relative risk reduction (RRR)	69.39%
Absolute risk reduction (ARR)	13.88%
Number needed to treat (NNT)	721

## Discussion

Currently, primary insertion of a voice prosthesis simultaneously during total laryngectomy has become the gold standard in voice rehabilitation. Originally, replacement of the voice prosthesis was performed in the event of a leak or the presence of other complications related to its use. After the method became more widely available and more accessible, questions about the optimal approach arose. In particular, trends in implantable devices such as pacemakers promote regular assessment and planned replacement, protecting against sudden dysfunction. A similar line became the basis for considering the regular replacement of voice prostheses as a routine protocol (9). However, no randomized clinical trials have evaluated the benefits and conditions for such recommendations thus far.

Our study comprehensively assessed the effectiveness of post-TL care in patients with voice prostheses. Patients were randomly divided according to the scheme of voice prosthesis replacement. Both groups were consistent in terms of demographic factors, with the exception of age. Despite random assignment to groups, LEA patients were significantly younger (p = 0.002). The two groups did not differ significantly in terms of clinical factors (primary treatment and adjuvant treatment) or smoking and alcohol consumption habits.

Comparative analysis of REA and LEA revealed a significantly longer time between replacements in the LEA group (p = 0.023) and a significantly lower rate of complications in the REA group (p = 0.029). These results are consistent with the project assumptions.

This study further analyzed the factors affecting the longevity of voice prostheses. In the LEA group, the average lifespan was 5.37 ± 4.74 months. The device lifetime varies in the literature, but most studies report an average period of 3–4 months (7, 10–13); however, the lifetime of prostheses ranges from 1–42 months (14). Owing to the presence of different types and brands of voice protheses, different device lifespans can be observed in different populations (15). Our results can therefore be generalized to all voice prosthesis patients worldwide.

Adjuvant therapy significantly influenced the longevity of voice prostheses (p = 0.032), with the longest device lifetime observed in patients after RCT (10.1 ± 5.15 months). However, this study included only 4 patients, so the sample size was too small to draw final conclusions. In addition, this result should be interpreted with extreme caution because one of the patients was on chronic parenteral nutrition and the other was on a strict diet due to a history of gastrectomy and intestinal surgery. These two patients may not require frequent prosthesis replacements due to the limited contact of the voice prosthesis with standard foods and saliva. Saliva components negatively affect the longevity of voice prostheses, as proven in our previous study (16).

The analysis of other studies indicates that RT and RCT should shorten the time between replacements (10, 17–19) because RT predisposes patients to enlargement of the tracheoesophageal fistula (17). However, recent reports contradict this hypothesis, indicating that RT has no effect on device lifetime (5, 7, 20–22). It is now postulated that the enlargement of the tracheoesophageal fistula is not related to RT but rather to increased vocal effort and friction between the prosthesis and the party wall due to a prosthesis that is too long and increased air pressure toward the fistula when muscle hypertonicity is present (5).

Moreover, the presence of concomitant diseases, dental conditions, diet or alcohol consumption did not affect the longevity of the voice prostheses. Soukka et al. reported that a diet rich in dairy products and probiotics has a positive effect on the longevity of vocal prostheses by reducing the colonization of *Candida albicans* (14). Enzymes produced by fungi influence the silicone biomaterial of the voice prosthesis (23).

Periprosthetic leakage was the most common complication associated with the use of voice prostheses, occurring in 3.06% of patients in REA and 9.09% in LEA. The difference in leakage rates was statistically insignificant (p = 0.137). Data on the percentage of leaks around the voice prosthesis are inconsistent (24). According to a systematic review by Hutcheson et al., between 3% and 11% of voice prosthesis replacements resulted in peri-prosthesis leakage. A study by Lorenz et al. revealed periprosthetic leakage in 35.7% of patients (20). Other studies reported leakage around the prosthesis in 21–24% of patients (5, 25). Thus, the percentage of leakages around voice prostheses in our study group was relatively small compared with the results reported in the literature. However, the percentage of patients with periprosthetic leakage was not significantly different between REA and LEA. In each of these cases, the patient was hospitalized, and a voice prosthesis was removed and inserted after several days of conservative treatment.

However, the present study indicates that the regular replacement of voice prostheses does not provide other measurable advantages over irregular replacement. Regular prosthesis replacements may prevent unexpected leaks and aspiration, improving patients’ quality of life (9). Prospective analysis of the two approaches to patient care did not confirm these advantages. Although the relative reduction in the complication rate was almost 70%, the absolute reduction was only 14%, and an additional 721 replacements should be performed in the preventive replacement scheme to avoid one complication. In addition, the variability in the device lifetime in LEA is high (intrapatient CV 53.65%, interpatient CV 65.79%), so it may be misconceptive to propose the same care to all patients. Replacing the voice prosthesis every three months in the majority of patients might generate unnecessary hospital visits and additional costs.

Apart from complication rates, regular care should provide patients with a sense of security and greater satisfaction; however, our study failed to prove that. Satisfaction with the use of the voice prosthesis was similar regardless of the regularity of replacement (p = 0.389). Furthermore, the regularity of replacement did not affect the difficulty of replacing the prosthesis or voice quality (p = 0.9 and 0.337, respectively). Furthermore, the advantage of regular exchanges is the possibility of organizing the work of hospital staff in such a way as not to affect the daily care of other patients. Before each replacement of voice protheses, patients are examined by a doctor, which provides the opportunity to consult current health problems and allows rapid detection of features that may indicate a recurrence of cancer. However, considering all the aspects presented, we believe that regular prothesis replacements are not cost-effective and do not provide tangible benefits to patients from a group-wide perspective.

The limitation of the present study is the relatively small number of included patients. Moreover, despite the random assignment to both arms, we did not avoid inconsistency in demographic terms between groups with significantly older patients in REA vs LEA (p=0.002), which could influence the results.

Continuation of multi-center studies and analyses on a large group of patients will allow for the development of more precise recommendations.

## Conclusions

This is the first prospective, randomized study to evaluate the benefits of regular and leakage-related replacement of voice prostheses in laryngectomy patients. Regular voice prosthesis replacement has advantages in terms of overall complication rates compared with the scheme based on leakage demand; however, it does not yield superior gains in terms of patient satisfaction or economic aspects.

## Data Availability

The original contributions presented in the study are included in the article/supplementary material. Further inquiries can be directed to the corresponding author.
